# Overweight effect on spirometric parameters in adolescents undergoing exercise

**DOI:** 10.1590/S1679-45082016AO3612

**Published:** 2016

**Authors:** Rayana de Oliveira Costa, Juliana Pereira Silva, Eliana Mattos Lacerda, Rodrigo Dias, Vitor Alexandre Pezolato, Carlos Alberto da Silva, Kleverton Krinski, Marco Aurélio de Valois Correia, Fabrício Cieslak

**Affiliations:** 1 Universidade Federal do Vale do São Francisco, Petrolina, PE, Brazil.; 2 Universidade Metodista de Piracicaba, Piracicaba, SP, Brazil.; 3 Universidade Federal de São Carlos, São Carlos, SP, Brazil.; 4 Universidade de Pernambuco, Petrolina, PE, Brazil.

**Keywords:** Obesity, Bronchial spasm, Adolescent

## Abstract

**Objective:**

To evaluate effects of overweight on spirometric parameters in adolescents who underwent bronchial provocation test for exercise.

**Methods:**

We included 71 male adolescents. The diagnosis of asthma was done based on participants’ clinical history and on the International Study Questionnaire Asthma and Allergies in Childhood, and the diagnosis of obesity was based on body mass index above 95th percentile. The bronchospasm induced by exercise was assessed using the run-walk test on a treadmill for eight minutes. The decrease in forced expiratory volume in one second > or equal to 10% before exercise was considered positive, and to calculate the intensity in exercise-induced bronchospasm we measured the maximum percentage of forced expiratory volume in one second and above the curve area. Data analysis was carried out using the Mann-Whitney U test and Friedman test (ANOVA), followed by Wilcoxon test (p<0.05). In addition, we used Fisher’s exact test to analyze the exercise-induced bronchospasm frequency.

**Results:**

Significant differences were observed among obese adolescents in exercise-induced bronchospasm frequency (p=0,013) and in relation to time required for recovery after exercise (p=0,007).

**Conclusion:**

Overweight can influence the increase in the exercise-induced bronchospasm frequency in non-asthmatic adolescents compared with eutrophic adolescents.

## RESUMO

**Objetivo:**

Avaliar o efeito do excesso de peso sobre parâmetros espirométricos em adolescentes submetidos ao teste de broncoprovocação por exercício.

**Métodos:**

Participaram do estudo 71 adolescentes do sexo masculino. O diagnóstico de asma foi obtido por meio de histórico clínico e do questionário *International Study of Asthma and Allergies in Childhood*, e o de obesidade, pelo índice de massa corporal acima do percentil 95. Para avaliar o broncoespasmo induzido pelo exercício, utilizou-se o teste correr/caminhar em esteira ergométrica, com duração de 8 minutos, considerando positivo se diminuição do volume expiratório forçado no primeiro segundo >10% do valor pré-exercício e, para a intensidade do broncoespasmo induzido pelo exercício, foram utilizados o cálculo da queda percentual máxima do volume expiratório forçado no primeiro segundo e a área acima da curva. A análise dos dados foi realizada pelo teste U Mann-Whitney e pela ANOVA de Friedman, seguido do teste de Wilcoxon (p<0,05). O teste de Fisher foi empregado para analisar a frequência de broncoespasmo induzido pelo exercício.

**Resultados:**

Foram encontradas diferenças significativas quanto à frequência de broncoespasmo induzido pelo exercício (p=0,013) e ao tempo de recuperação pós-exercício (p=0,007) nos adolescentes obesos.

**Conclusão:**

O excesso de peso pode influenciar no aumento da frequência de broncoespasmo induzido pelo exercício em adolescentes não asmáticos, quando comparados a eutróficos.

## INTRODUCTION

Obesity is a chronic non-transmissible disease, characterized by accumulation of body fat, from genetic, biological, environmental, psychological and sociocultural interactions. Obesity is considered a global epidemic and public health problem, which is justified by its high incidence, in Brazil, specifically, it reaches about 15% of child and youth population. For this reason, this disease presents significant affection by associated comorbidities.^(1,2)^

Studies suggest that fat tissue accumulation in the organism has the potential to increase cardiovascular risk, predisposition to type 2 *diabetes mellitus*, respiratory diseases, and musculoskeletal disorders.^(2-4)^ Obesity specifically associated with respiratory dysfunctions is directly related to systemic pro-inflammatory status generated by mechanical ventilation^(5-7)^ that may cause increase of bronchial hyperreactivity, therefore indicating that overweight contributes to progression of exercise-induced bronchospasm (EIB).^(8)^

The EIB is characterized by temporary obstruction of airways after exercise, resulting in a reduction of forced expiratory volume in the first second (FEV_1_) to values higher than 10% compared with pre-exercise values. Its prevalence occurs in approximately 5 to 20% of population not diagnosed with bronchial asthma, and its intensity appears to be higher in children and adolescents, especially in those overweight.^(9,10)^

Physical exercise has been constant adopted as a preventive/therapeutic method to reduce body mass (BM), in addition it improves cardiorespiratory fitness. However, recent studies have associated obesity with bronchial hyperreactivity and respiratory symptoms after exercises in adolescents, which result in more resistance for physical exercise and, also, maintaining and worsening overweight, with important limitation of pulmonary function and experience of related disorders.^(5,7)^

Considering respiratory symptoms as one of determining factors for resistance to physical activity in obese individuals, it became extremely necessary to better understand this possible limitation for exercise with the aim to reduce high rates of sedentarism and obesity in the world.^(10,11)^ Experts believe that achieve higher frequency of EBI in overweight adolescents is something feasible.

## OBJECTIVE

To evaluate effects of overweight on spirometric parameters in adolescents who underwent exercise bronchoprovocation test.

## METHODS

The sample included 71 adolescents aged 12 and 16 years recruited at a public school in the city of Petrolina (PE), Brazil, from July to September 2015.

After anthropometric screening, adolescents were divided into two groups: Overweight Group (OWG) including 43 individuals, and Eutrophic Group (EG) with 28 individuals. Participants were classified based on body mass index (BMI), following criteria defined by the Center for Disease Control and Prevention (CDC). Tests were collected by the Human Development Research Laboratory of the *Universidade do Pernambuco*.

The number of participants was calculated based on level of significance of 0.05 and statistical power of 0.90, and magnitude of high effect (ƒ2=0.80) according to classification established by Cohen^(12)^ that defined a minimal number of 28 participant for each group. Subsequently, we designed a method for recruiting by convenience to invite possible participants.

Inclusion criteria to participate in all evaluations were: present signed consent form by parents or responsible; individuals classified as obese; self-report of no respiratory infection within 4 weeks before the test - based on medical tests performed before beginning of evaluations; self-report of any drug treatment, and no history of cardiovascular, respiratory, musculoskeletal and/or metabolic disorders. In addition, self-report of non-use of food or medications with caffeine within 2 hours before tests. We excluded individuals who were classified as pre-puberty in the assessment of sexual maturation.

Participants and/or their responsibles signed the consent form after receiving explanation about objectives, procedures and possible risks of the study. This study approved by the Ethical and Deontology Committee in Studies and Researches of the *Universidade Federal do Vale do São Francisco*, register 0009/131113.

The BM, in kilograms, was measured using a digital balance (Plenna^®^, Brazil) with a precision of 100g and maximum capacity of 150kg. Participants’ body weight was taken with individual remaining at center of the platform in orthostatic position, without shoes, with arms alongside the body and wearing light clothes. Participants height, in centimeters, was checked using a wall mounted stadiometer (Sanny^®^, São Paulo, Brazil) standard, with precision of 0.1cm with participant on orthostatic position, feet together and without shoes, still in apnea respiratory, head on Frankfort horizontal plane, and with posterior surfaces of the calcaneal, pelvis waist, scapular waist, and occipital region on touch with the measurement instrument.^(13)^

From participants’ BM measures and heights, we calculated the BMI, which was posteriorly classified according to percentages for normality (between 5° and 85°) and overweight (between 85° and 95°), according to sex, age and ethnicity.^(14)^

Sexual maturation was evaluated based on Tanner’s criteria.^(15)^ To emphasize the diagnosis of asthma, the questionnaire of International Study of Asthma and Allergies in Childhood (ISAAC) was applied to all participants.^(16)^

To evaluate pulmonary function, participants were oriented to not drink coffee, tea or soft drink with caffeine 2 hours before the test, and not use bronchodilators of short and long action 12 hours before, and suspend the anti-histaminic of short and long action, respectively, 48 hours and 5 days before the assessment. Patient could not also present symptoms of upper airways viral infection within the last 4 weeks.

Pulmonary function was measured from variables of forced vital capacity (FVC) and FEV_1_, both expressed in liters. For this measurement a spirometer (Cosmed, Microquark, São Paulo, Brazil), was used. The individual was maintained seated and a nasal clip was used during measurement. We conducted three spirometric maneuvers, and selected those with more values of FEV_1_ and FVC for age, sex, height and weight, based on Polgar et al.^(17)^ FEV_1_ and FVC.

Bronchoprovocation test with exercises such as running/walking were done using an ergometric treadmill (Master Super ATL, Inbramed^®^, São Paulo, Brazil), using official guidelines from the American Thoracic Society with enough intensity to achieve 80% to 90% of maximum heart rate (maxHR), previous calculated in the first 2 minutes and maintained for 6 minutes.^(18)^ Heart rate (HR) was monitored using a Polar^®^ heart rate monitor before, during and after the test. Treadmill inclination was established in 10%,^(18)^ and initial speed was estimated by the following equation:^(19)^


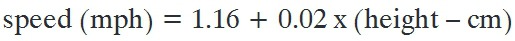


Tests were carried out in morning, 8 a.m. to 11 a.m., with controlled environmental conditions using a digital hygrometer (Perception II, Davis^®^, São Paulo, Brazil), maintaining the temperature between 20 and 25°C and relative humidity of air between 40 and 50%.

Pulmonary function after test was evaluated through FEV_1_, in liters, in 5, 10, 15 and 20 minutes after physical exercise. The EIB was considered positive for reduction of FEV_1_ ≥10% to pre-exercise value, according to previous studies.^(9,19)^

After physical exercise, we calculated the maximal percentual fall of FEV_1_ (%MFFEV_1_), using calculation of percentage decreasing of FEV_1_ after exercise in relation to pre-exercise value by the following equation:


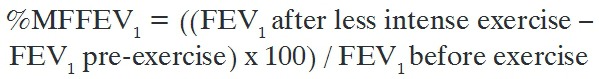


Area above curve (AAC_0-30_) was obtained by trapezoid model proposed by Prince,^(20)^ to analyze intensity of EIB during all the interval of time for recovery in period after test.

For data analysis, we employed descriptive statistics with use of frequencies, percentages, media and standard deviation to identify student’s characteristics in the study. To verify data we used the U Mann-Whitney test and Friedman’s analysis of variance (ANOVA), followed by Wilcoxon test. In addition, we used Fisher test to analyze frequency of EIB. Data were analyzed using Statistical Package for the Social Sciences (SPSS) software, version 21.0 for Windows, with level of significance estimated in p<0.05 for all analyses.

## RESULTS

For comparison of anthropometric variables we identified similarity between OWG and EG in relation to initial characteristics (Table 1).

**Table 1:** Anthropometric data

Characteristics	OWG (n=43)	EG (n=28)	p value
Age (years)	12±1.33	12±1.38	0.13
Weight (kg)	67±8.05	45.5±8.24	0.04*
Height (cm)	160±8.1	155±9.71	0.15
BMI (kg/cm^2^)	26.9±1.56	18.15±1.88	0.02*

*p<0.05.

OWG: Overweight group; EG: Eutrophic group; BMI: body mass index.

**Figure 1:**
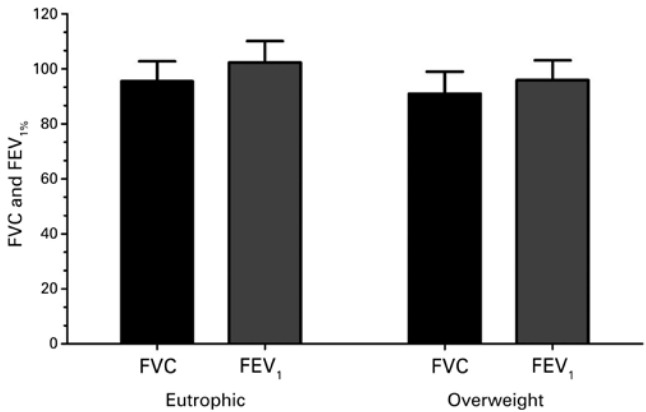
Comparison of forced vital capacity and forced expiratory volume in the first second between groups before exercise

Resting lung function was evaluated using FEV_1_ and FVC. There were similarities between groups, but we observed an increase of BMI in the OWG that was associated with reduction of function (Figure 1).

FVC: forced vital capacity; FEV _1:_ forced expiratory volume in the first second.

**Figure 2:**
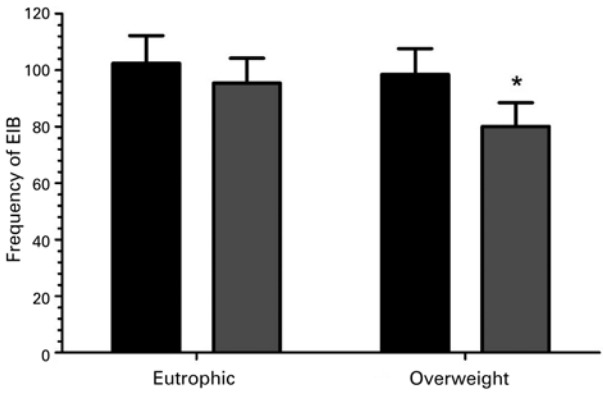
Frequency of exercise-induced bronchospasm between groups. Data are reported in mean ± standard deviation

The EIB was positive in eight adolescents from the EG, corresponding to 28.6% of the group, and 31 adolescents from OWG corresponding to 72.1% of the group. We observed difference in frequency of EIB between groups (p=0.013) (Figure 2).

*Fisher test p=0.013 (p=0.05).

EIB: exercise-induced bronchospasm.

**Figure 3:**
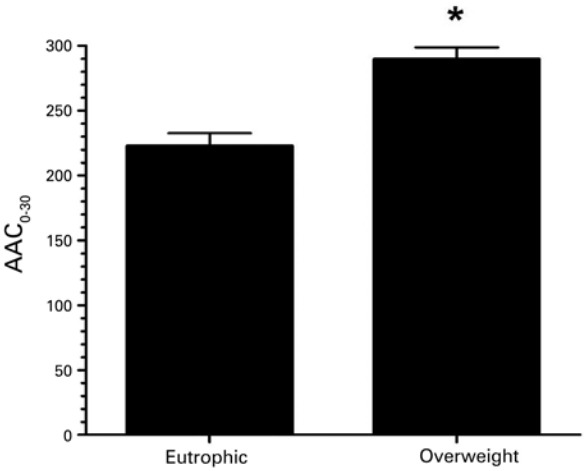
Comparison between area above curve (AAC0-30) values in eutrophic and Overweight Group. Data are expressed in mean ± standard deviation

Values of AAC_0-30_ that represented the interval between maximal fall of FEV_1_ and recovery time were significantly higher in the OWG (p=0.007) (Figure 3).

*Friedman’s test p=0.007 (p=0.05).

**Figure 4:**
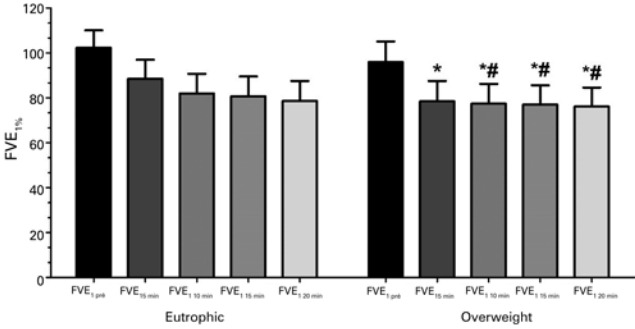
Recovery time after exercise (5, 10, 15 and 20 minutes) between groups

In relation to recovery time, including the 20 minutes after test, we observed significant differences between OWG and EG (p=0.008) and also in relation to interval among them, in pre-exercise at 10 (p=0.009), 15 (p=0.008) and 20 minutes (p=0.007) (Figure 4).

*Fisher test p=0.013 (p=0.05); *# Mann-Whitney test, sequentially, 0.009, 0.008 and 0.007 (p=0.05), in relation to FEV_1_ pre and FEV_1_ 10min, FEV_1_ 15 min and FEV_1_ 20 minutes.

FEV_1_: forced expiratory volume in the first second.

## DISCUSSION

Overweight promotes changes of mechanical proprieties of respiratory system, mainly for excess chest fat, limiting the lung expansion, in addition to impair strengths of dilation that maintain the potency of airways and which lead, possibly, the increase of contractility and responsiveness on smooth musculature of airways. In addition, fat tissue is an important source of pro-inflammatory cytokines and chemokines, and high levels of these mediators influence in changes of inflammatory response to airways.^(10,21-23)^

Initial characteristics were similar among groups, and it was possible related to development stages of evaluated individuals who had already reached puberty. Overweight adolescents would be in the front related to sexual maturity and stature.^(22,23)^ However, this variable provided us data only to promote standardization among participants.

Accumulation of adiposity can generate changes in respiratory mechanic leading to reduce in volumes and lung capacity. However, the pulmonary function is similar between obese and non-obese children and adolescents.^(6)^ This study observed that there was no significant difference in FEV_1_ and FVC at resting among overweight and eutrophic adolescents. However, with prevalence of obesity more early, changes in respiratory mechanism can be seen even in childhood,^(4,7,8)^ as shown in this study.

Obese individuals often report dyspnea and respiratory distress and physical efforts, which suggests EIB.^(23,24)^ However, to confirm this feature, it is necessary to evaluate lung function from the FEV_1_ behavior after exercise.

Studies have found significant reduction of FEV_1_ after exercise in obese children and adolescents compared with non-obese children and adolescents,^(3,4)^ and similar results, when compared with asthmatic.^(22)^ In our study, the OWG had maximal fall of FEV_1_ significantly higher than EG.

The area above the curve represents the fall and recovery of FEV_1_ to values pre-exercise. Few studies considered this variable to evaluate lung function of obese children and adolescents.^(4)^ In our study, we observed a trend of OWG in present a higher area in comparison to EG.

Percentage values FEV_1_ fall, which characterizes EIB, differed among researchers. Studies suggest a fall of ≥15%; others suggest that a fall of 10% is representative of bronchoconstriction importance.^(25-27)^ In this way, the frequency of EIB between different studies carried out must be careful checked.

Studies that investigated frequency of EIB among obese and non-obese children and adolescents found conflicting results. Based on this fact, Cieslak et al.^(4)^ investigated the effect of obesity in spirometric parameters in adolescents submitted to bronchoprovocation test by physical exercise. We evaluated 15 obese and non-obese adolescents of both sexes. Results observed moderate negative relationship for analyzed variables (%QMFEV_1_ e AAC_0-30_). On the other hand, Ulger et al.^(7)^ found a significant higher frequency in obese compared with non-obese individuals. In our study, we used a fall of 10% of FEV_1_ with reference values to evaluate frequency of EIB. Using this approach, we obtained frequencies for OWG in relation to EG, revealing that accumulation of fat tissue can be considered a limiting factor to trigger EIB.^(26,28)^

Therefore, vigorous exercise can provide bronchoconstriction in overweight adolescents, but inactivity or reduction of physical activity can be accept due to gains for health and life quality of individuals, since they are medicated and adequately followed-up.^(26)^

This study limitation is related to evaluation of sexual maturity, which was used only to characterize the sample. In studies in the future, sexual maturity can be included in evaluation in order to provide better understand of results related with hormonal effects.^(10)^

## CONCLUSION

In general, we can consider that overweighed adolescents had higher fall of maximal percentage of forced expiratory volume in the first second when compared to eutrophic adolescents. In addition, recovery time and behavior of forced expiratory volume in the first second after exercise were related to systemic inflammatory status generated by obesity to systemic inflammation in obesity. However, further investigations are need to confirm this parallel and also that can control limitations identified in this analysis.
